# Supplementary figures and images for: Closely related reovirus lab strains induce opposite expression of RIG-I/IFN-dependent versus -independent host genes, via mechanisms of slow replication versus polymorphisms in dsRNA binding σ3 respectively
Source: PLoS Pathog. 2020 Sep 21;16(9):e1008803. doi: 10.1371/journal.ppat.1008803 (PMC7529228; doi:10.1371/journal.ppat.1008803)

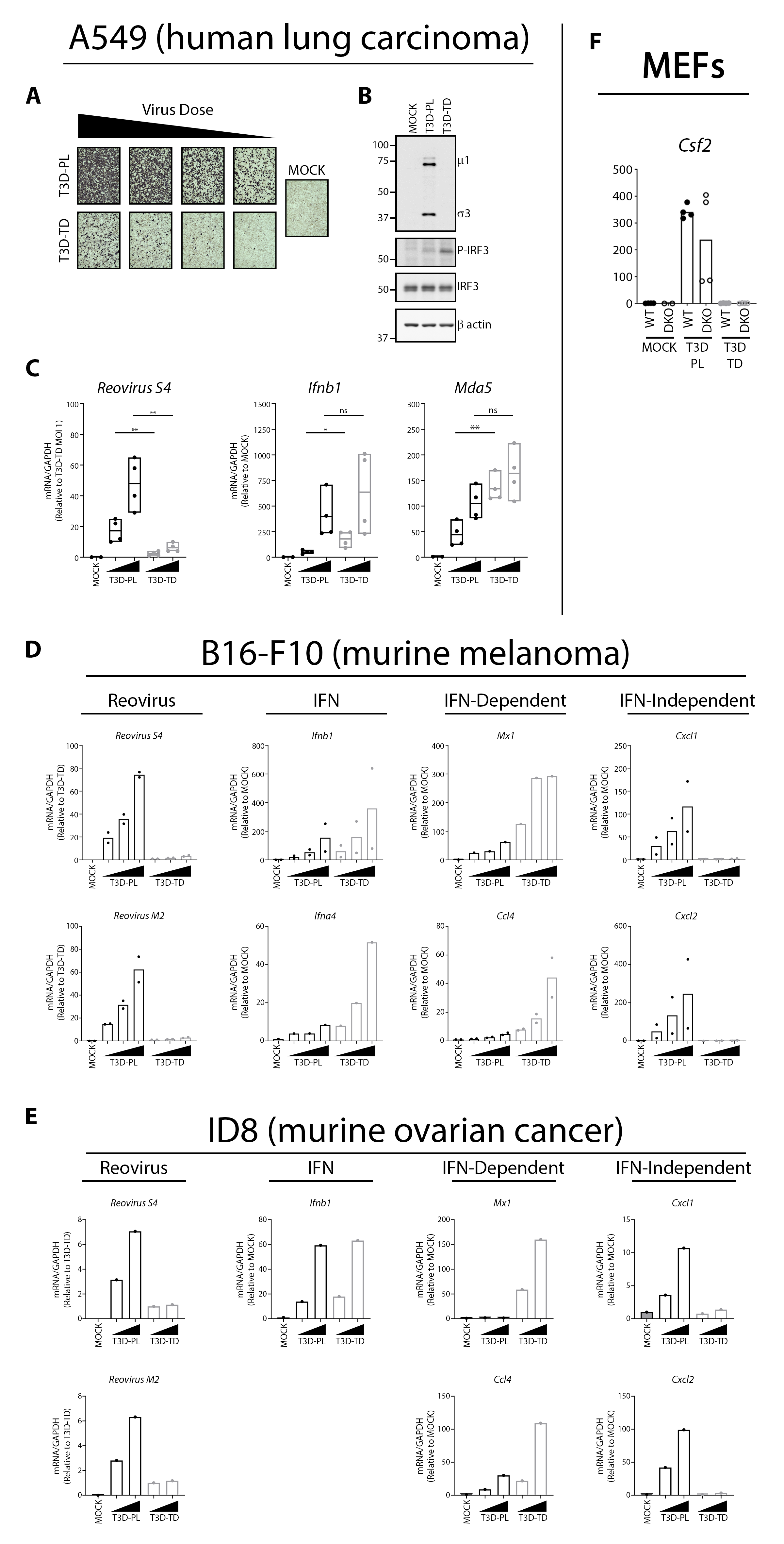

Supplement: S1 Fig — (A-E) Cells were infected with T3DPL or T3DTD and samples were collected at 12hpi for immunocytochemical staining of reovirus infected cells (A), Western blot analysis of reovirus proteins (μ1, σ3), IRF3, P-IRF3, β actin (B) or RNA extraction followed by cDNA synthesis and RT-qPCR for the indicated genes (C-E). (A-C) A549 cells. (D) B16-F10 cells. (E) ID8 cells. All genes except Reovirus S4/M2 were normalized to MOCK. Reovirus S4/M2 values were normalized to the lowest T3DTD dose. Each point represents a biological replicate n = 1–2. (F) Similar to Fig 5A, RIG/IFN-independent Csf2 mRNA levels relative to housekeeping gene GAPDH was quantified using RT-qPCR, in WT or RIG-I/MDA5 -/- double knockout (DKO) MEFs infected with T3DPL or T3DTD at MOI 6 for 12hpi. Values were normalized to MOCK WT MEF. Each point represents a technical replicate for n = 2 independent experiments. (TIF) [file ppat.1008803.s001.tif]

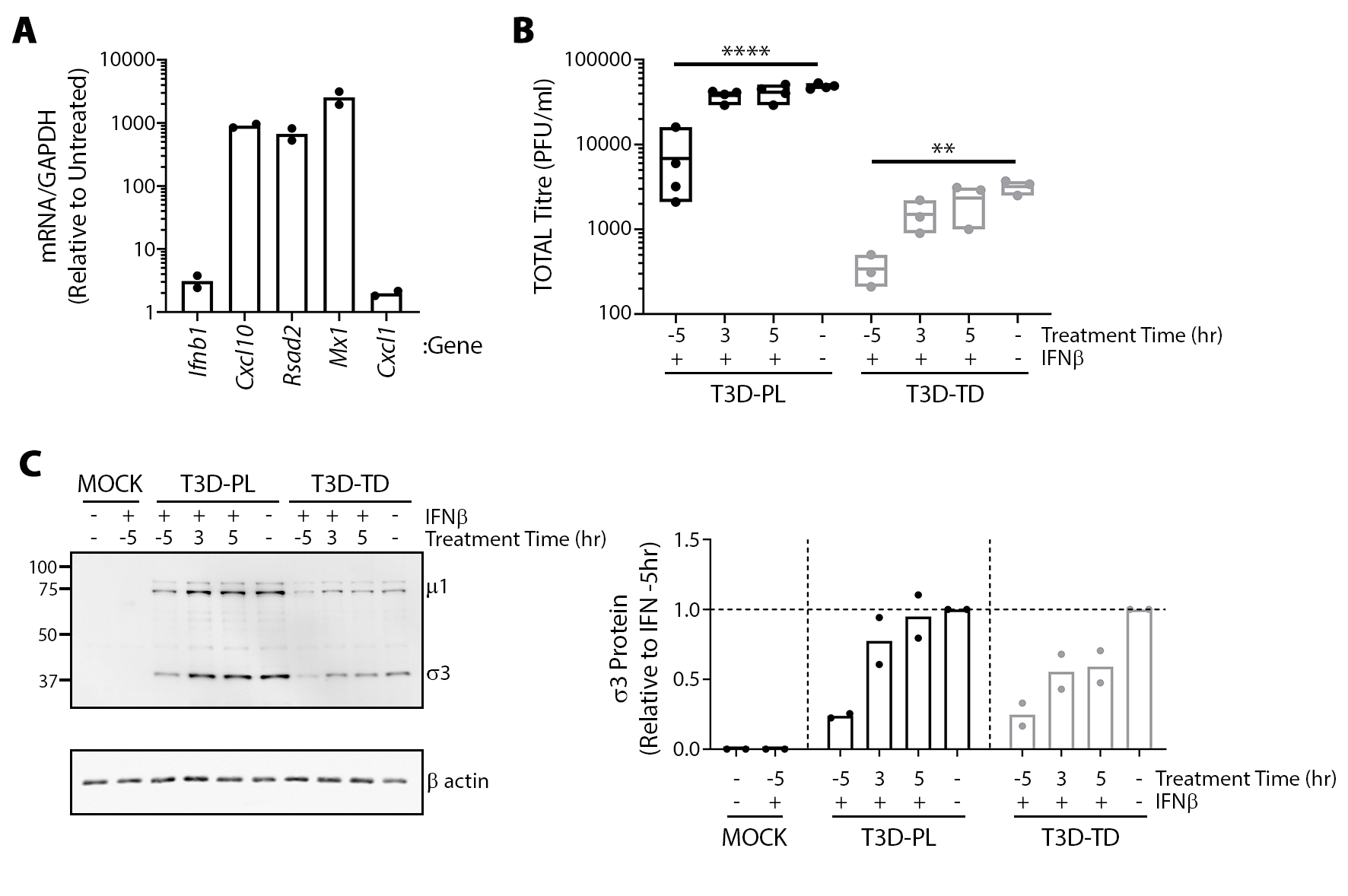

Supplement: S2 Fig — (A) L929 cells were treated with 1000 U/ml/12well of purified IFNβ for 18hrs at 37°C. Samples were collected for RNA extraction, cDNA synthesis and RT-PCR using gene-specific primers (corrected for GAPDH). Values were standardized to untreated sample. (B-C) L929 cells were treated with IFNβ at the indicated timepoints and/or infected with T3DPL or T3DTD for 18hrs. Samples were collected and processed for viral titres (B) and Western blot analysis (C). Protein samples in (C) were quantified using densitometric band analysis with an ImageQuantTL. Each point represents a biological replicate. (TIF) [file ppat.1008803.s002.tif]
