# Supplementary material for: Closely related reovirus lab strains induce opposite expression of RIG-I/IFN-dependent versus -independent host genes, via mechanisms of slow replication versus polymorphisms in dsRNA binding σ3 respectively
Source: PLoS Pathog. 2020 Sep 21;16(9):e1008803. doi: 10.1371/journal.ppat.1008803 (PMC7529228; doi:10.1371/journal.ppat.1008803)
Supplement: Attachment — Submitted filename: Response to Editors and Reviewers.pdf [file ppat.1008803.s004.pdf]

EDITORS NOTES:

1. [Original Editor comment] It is possible that variation in the particle:PFU ratios between virus preps could influence IFN induction. This was of particular importance to the testing of the reassortant viruses.

[Editor response to our request for revisions] If the particle:PFU ratios were not known, there is a high likelihood that all of the experiments in figure 1 and 2 would need to be repeated. However, the authors apparently had that data in hand. Moreover, there is a normal level of variability between the stocks (at least for reoviruses) that I do not think would impact the results.

The particle:pfu ratios for T3D^{PL} and T3D^{TD} parental viruses and respective S4, M1 and L3 gene reassortant viruses were calculated and have been included as a part of Figure 2A. Accompanying methods for determining particle:pfu ratios have been included in the materials/methods section. Importantly, we have elaborated to explain that pfu:particle ratios have minimal impact on IFN induction by reassortant viruses with respect to the parental viruses. Specifically, Lines 188-195 state: "To explore the possibility that differential levels of defective virions in the virus preparations could impact IFN induction, we compared specific infectivity of the viruses. The ratio of total particles versus infectious particles was determined using spectrophotometry at OD260 and standard plaque assay, respectively (Figure 2A, values below plaque photographs). There was no clear relationship between the specific infectivity and IFN induction (Figure 2A versus 2B); therefore, increased IFN induction could not be easily explained by an increased proportion of defective or non-productive virions. "

2. [Original Editor comment] There was no direct test of whether IFN priming underlies differences between the initial and subsequent rounds of replication by pretreating with IFN.
[Editor response to our request for revisions] The authors have some valid points, however going back to look at the data even more closely, the authors data suggests that IFN responses during the initial round of infection does affect impact the number of cells infected, but the viral burst is not measured. Thus, differences in the amount of virus produced between wild-type and RIG-I/MDA5 DKO cells in the first round of replication could the reason for the increase in the number of infected cells in subsequent rounds. It is also possible that IFN production by wild-type cells limits replication in newly infected rounds, as one reviewer suggested testing.

Additional experiments were completed and added to the manuscript to address this important point:

1. We now provide data showing that both viral mRNA levels (previously included), and now virus burst size (new data), are not increased in DKO cells, supporting further that IFN does not impinge on the first round of replication.

We now describe in lines 299-319: "A direct and physiologically relevant approach to implicate a pathway in virus replication is to remove the pathway and determine if virus replication is changed. Accordingly, we made use of double knock-out (DKO) mouse embryo fibroblasts (MEFs) lacking both RIG-I and MDA5 (41, 43). DKO cells were confirmed to show minimal induction of IFNs (Ifnα4) and IFN-induced gene Rsad2 following 12 hours of infection by either T3DPL or T3DTD (Figure 5A). We reasoned that if IFN signalling can impact the first round of virus infection, then the DKO cells should demonstrate increased infection by reovirus at an intermediate timepoint of 12-18hpi where cell-cell spread is minimal. Wild-type (WT) and DKO MEFs were exposed to equivalent infectious dose of T3DPL or T3DTD. The percent of cells productively expressing viral antigens was measured by flow cytometric analysis (Figure 5B) or visually monitored by fluorescence microscopy (Figure 5D, left). S4 reovirus mRNA production was quantified at 12hpi by RT-qPCR (Figure 5C). Virus input versus burst size were assessed by standard plaque titration (Figure 5D). By all three parameters of reovirus replication, when focusing on timepoints prior to cell-cell spread (≤18hpi), T3DPL outperformed T3DTD in the first round of infection as was anticipated. Most critically, knock-out of IFN production had minimal effects on either T3D strain during the initial round of replication. Note that IFN production did, however, exert the expected paracrine inhibitory effects on dissemination and subsequent rounds of reovirus replication, evident by increased numbers of infected cells (Figure 5B, 24hpi), titers (Figure 5D, 48hpi), and even plaque size (Figure 5E) in DKO relative to WT cells at time points ≥24hpi . The data was suggesting that T3DTD, via slower replication kinetics, causes greater IFN signalling; rather than that greater IFN signalling causes slower T3DTD replication kinetics. Moreover, while differences in IFN

signalling are not responsible for delayed replication kinetics of T3DTD, they are likely to favor enhanced cell-cell spread for T3DPL over multiple rounds of infection."

2. As suggested by a reviewer, we tested the effects of adding recombinant IFN to the media, both pre- and post-infection. This new experiment suggests that IFN only minimally exerts inhibitory effects on virus replication when added after virus entry and uncoating is complete.

Lines 340-352 now describe: "As a third complementary strategy to assess the impact of IFNs on the first round of T3D reovirus replication, we treated L929 cells with IFNβ at various timepoints pre- and post- reovirus infection, and monitored cytokine expression (Figure S1A), virus titers (Figure S1B), or virus protein expression (Figure S1C) at 18hpi. We first validated that IFNβ treatment for 5hrs, in absence of virus, causes potent activation of prototypic ISGs (Cxcl10, Rsad2, Mx1), but not non-IFN-induced cytokines Ifnb1 itself and Cxcl1 (Figure S2A). In congruence with the paracrine effects of IFNβ on suppressing reovirus cell-cell spread in Figure 5, when cells were pre-treated with IFNβ for 5hrs prior to reovirus exposure (-5), there was strong inhibition of burst titers (Figure S1B) and protein synthesis (Figure S1C). To mimic potential autocrine effects of IFNβ on already-infected cells, recombinant IFNβ was added 3 or 5 hours post reovirus exposure. Effects of IFNβ on viral protein and titer accumulation was clearly diminished over time of infection, with minimal (not significant) effects by 3-5 hours post-infection for both T3DPL and T3DTD. Therefore, addition of IFNβ at timepoints following reovirus entry and uncoating had nominal impact on virus replication."

3. [Original Editor comment] It could not be discerned whether the cells in co-infection experiments were actually infected by by both viruses.
[Editor response to our request for revisions] I agree with the authors that the high MOI likely ensures co-infection. However, RNAs from both viruses were not detected in co-infected cells using high-resolution melt analysis. This could be due to technical limitations or potentially the large differences in replication between the co-infecting strains. However, the authors go into detail about the sensitivity of the assay and it appears that their analysis should be able to detect both viruses based on the levels shown in Fig. 4F. Consequently, I thought the reviewer comment valid.

We address this concern by extending our description of the virus dose and our controls to ensure true multiplicities of infection. Specifically, lines 255-263 now state: "To validate this unexpected finding, we therefore repeated co-infections at increasingly higher MOIs, and took extra steps to confirm that doses were more-than-sufficient to infect all cells. L929 cells were exposed to T3DPL, T3DTD, or both strains at MOIs of 20, 60, and 180. Viruses were permitted to bind to cells at 4oC for 1 hour, washed extensively, and then western blot analysis was performed to confirm that similar cell-bound virus particles were detected for T3DPL and T3DTD (Figure 4B). The infection was then allowed to proceed at 37oC for different timepoints. At 12hpi and 24hpi, the number of reovirus antigen-expressing cells was measured by flow cytometry (Figure 4C). As seen in Figure 1C, T3DTD infection was delayed relative to T3DPL, but importantly, copmlete infection of all cells was achieved by both T3D strains."

We ask the editor and reviewer to please consider that co-infection can be equated from the basic definition of MOI as "multiplicity of infection", meaning infectious units per cell, and then gaussian distribution (e.g. MOI of 3 gives approximately a majority of infected cells, which we show throughout the manuscript). No evidence for supression of co-infection has been shown for reovirus, and there is no study of reovirus co-infections that I can find, where it is questioned whether such high MOIs are sufficient for co-infection. Also although not present as data in this manuscripts, we readily obtain reassortants using MOIs of 10.

4. [Original Editor comment] Related to the co-infection experiments, how does establishment of separate replication centers by fast- and slow-replicating viruses allow for reassortment?
[Editor response to our request for revisions] The authors' point is valid and discussion of it should be included in a revised version.

This is a good and fun question. The virions that enter a cell can establish separate replication centers around them, but ultimately everything moves towards the perinuclear region where all viruses have opportunity to mix. We are now promising a new idea based on our data, that during the initial "starting" replication centers, the incoming virions can exhibit distinct effects on the same cell depending on the genetics of the incoming virus. So, in a co-infection, one strain of reovirus can induce RIG/IFN signaling while the other does not. Yet eventually, both strains will mix and can re-assort; but the mixing is besides the point of RIG-I/IFN induction and beyond the scope of this manuscript.
We now address this in the discussion, specifically lines 535-541 describe ". It should be noted however, that virus factories/inclusion bodies eventually converge into a large perinuclear body; since we and others can easily generate reassortants during co-infection, the overall model needs to consider that incoming strains, although exerting distinct effects on IFN induction early, eventually mix and exchange segments."

5. [Original Editor comment] The relationship to the oncolytic properties of reovirus is a lynchpin of the rationale for the study and key to the broader impact of the work. However, the vast majority of the results were obtained in L929 cells and the relevance of the results to oncolysis is unclear.
[Editor response to our request for revisions] L929 cells are derived from normal fibroblasts, although they are immortalized. However, they are not generally considered to be transformed cell. The comment is from a reviewer that was specifically selected because they are an expert on cancer and oncolytic viruses, so I am inclined to trust their opinion. The authors claim to have performed many of the experiments in cell lines that are considered transformed and could be included. My recommendation was based on the likelihood that new experiments would be required. However, if the experiments have been performed in other cell lines I think they should be included as supplemental figures. This would alleviate the reviewer concern and enhance the broader implications of the work.

Although L929 cells are typically referred to as immortalized normal fibroblasts, they were characterized as tumorigenic in mice [1-3], are highly permissive to reovirus, but importantly were previously used in our panel of human and mouse cancer cells lines and represented common phenotypic differences between T3D strains [4, 5]. Nevertheless, we completely agree that panels of cancer cells are required to understand the applicability of conclusions to diverse cancer cell backgrounds; and therefore we now attached our additional data showing reproducibility of key findings from our study in A549 cells (human lung carcinoma), B16-F10 (murine melanoma) and ID8 (murine ovarian cancer). Importanty, our previously demonstrated differences of T3D strain oncolysis in vivo used the B18-F10 melanoma model [4, 5], so the data we currently show in B16-F10 cells has extended relevance.

Lines 144-146 now describe L929 better: "Phenotypes were first characterized in tumorigenic L929 mouse cells (31-33), since our previous comparisons found that enhanced oncolytic properties of T3DPL in L929 cells was representative of a panel of human and mouse cancer cell system (19, 20). "

Lines 164-168 describe other cancer cell lines: "Increased phosphorylation of IRF3 by T3DTD relative to T3DPL was reproduced in A549 human lung carcinoma cells (Figure S1A-C). Increased induction of IFN/ISGs by T3DTD was further confirmed in ID8 murine ovarian cancer cells, and in the B16-F10 murine melanoma system where T3DPL was previously characterized to exert more oncolytic activity in vivo relative to T3DTD (Figure S1D, E)."

Lines 410-413 also describe other cells that reproduce the key conclusions: "Most surprising about the findings, is that RIG-I/IFN-dependent versus independent signalling were inversely activated by T3D laboratory strains, which held true in five cell lines evaluated: NIH/3T3 (Figure 7C, n=2), L929 (Figure 7D, n=3), MEF (Figure 5A versus S1F, n=2), B16-F10 (Figure S1D , n=2) and ID8 (Figure S1E, n=1). The data indicates that reovirus strains can differentially active both IFN/RIG-I-dependent and independent genes, and that minor virus genomic diversity has potential to induce distinct host gene and cytokine profiles.

6. [Original Editor comment] The presentation of some data may be difficult for readers to interpret (see specific comments from Reviewer 3).
[Editor response to our request for revisions] Authors' comments acceptable.

Yes, we appreciate all of the minor suggestions by reviewers and have definitely addressed them (see specific answers to reviewers please for the corrections).

7. [Original Editor comment] Statistical analysis is missing from certain figures.
[Editor response to our request for revisions] Authors' comments acceptable.

As correctly suggested by reviewers, all figure legends now include details on the experimental replicates and specific statistical analysis. All key findings contain 3 or more independent experimental replicates with appropriate statistical analysis. Supportive findings using the shRNA knockdown model system were analyzed from 2 independent experimental replicates.

References cited in response

1. Rodriguez, T., Rengifo, E., Gavilondo, J., Tormo, B. & Fernandez, A. Morphologic and cytochemical study of L929 cell variants with different metastasizing ability in C3HA/Hab mice. *Neoplasma* **31**, 271-279 (1984).
2. Gavilondo, J. *et al.* Neoplastic progression evidenced in the L929 cell system. II. In vitro growth properties and biochemical characteristics of cell variants with different malignant behavior. *Neoplasma* **29**, 281-293 (1982).
3. Gavilondo, J., Fernandez, A., Castillo, R. & Lage, A. Neoplastic progression evidenced in the L929 cell system. I. Selection of tumorigenic and metastasizing cell variants. *Neoplasma* **29**, 269-279 (1982).
4. Mohamed, A. *et al.* Single amino acid differences between closely related reovirus T3D lab strains alter oncolytic potency in vitro and in vivo. *J Virol* (2019).
5. Mohamed, A., Smiley, J.R. & Shmulevitz, M. Polymorphisms in the most oncolytic reovirus strain confer enhanced cell attachment, transcription and single-step replication kinetics. *J Virol* (2019).
6. Broering, T.J. *et al.* Reovirus nonstructural protein mu NS recruits viral core surface proteins and entering core particles to factory-like inclusions. *J Virol* **78**, 1882-1892 (2004).
7. Broering, T.J., McCutcheon, A.M., Centonze, V.E. & Nibert, M.L. Reovirus nonstructural protein muNS binds to core particles but does not inhibit their transcription and capping activities. *J Virol* **74**, 5516-5524 (2000).

REVIEWERS COMMENTS AND CONCERNS

****
Reviewer's Responses to Questions

Part I - Summary</br>Please use this section to discuss strengths/weaknesses of study, novelty/significance, general execution and scholarship.

Reviewer #1: Overall, I think the findings presented by Adil Mohamed et al. are of sufficient novelty and interest for the RNA virology community at large. However, I do think that the paper would benefit from further clarifications and possible additional experiments. While I knowledge and value the hard-work from the authors, here are my comments, which are intended to further strengthen the manuscript.

Reviewer #2: The authors investigate the differential effects of two laboratory variants of the reovirus prototype strain type 3 Dearing (T3D) on host cell innate immune signaling. They demonstrate that despite slower replication kinetics (at both the RNA and protein level), the T3D-TD variant induces higher levels of IFN and ISG mRNA than the T3D-PL variant, based on polymorphisms in the S4, M1, and L3 gene segments. Experiments that decrease the replication kinetics of T3D-PL using inhibitors of RNA and/or protein synthesis result in enhanced IRF-3 phosphorylation, suggesting that the rate of replication the exposure of viral PAMPs to cellular pattern recognition receptors. Interestingly, in co-infection experiments, the T3D-TD strain was phenodominant, in that high levels of IRF-3 phosphorylation were observed, despite robust replication of T3D-PL in comparison to T3D-TD. The authors utilized either RIG-I/Mda-5 double knockout (DKO) fibroblasts or shRNA-mediated knockdown
of RIG-I to demonstrate that these pathways did not impact reovirus infectivity or early rounds of viral RNA or protein synthesis, despite their efficacy in reducing induction of IFNs and ISGs. The authors then conducted a whole genome microarray analysis in NIH3T3 cells over- or under-expressing RIG-I or under-expressing the IFN receptor. These experiments, when repeated in other cell lines, identified a number of IFNR-independent, RIG-I-independent genes that were upregulated by reovirus infection, and interestingly, this subset of genes were also differentially induced by the T3D strains, with T3D-PL inducing higher levels than T3D-TD (in contrast to the RIG-I/IFN-dependent genes). Finally, the authors confirm that polymorphisms in the S4 gene were responsible for the differences in expression of a subset of these RIG-I/IFN-independent genes.

Overall, the manuscript presents intriguing data highlighting the sharp differences in cellular responses that can be elicited by very closely related virus strains, which has significant implications for the use of these strains as potential oncolytic therapies. The data is generally clear and well-controlled and the manuscript is generally well written.

Reviewer #3: This study examined the differences in interferon (IFN) induction between two closely related isolates of T3D reovirus (T3D-PL and T3D-TD). Here it is shown that the PL isolate replicates quickly and lower levels of IFN and IFN-stimulated genes (ISGs) are detected in cells, whereas the TD isolate replicates more slowly and higher levels of IFN and IFN-stimulated genes are detected. Co-infection experiments demonstrated that the lower levels of IFN production in cells infected with the PL isolate did not appear to be due to a virally encoded IFN antagonist. The slower replicating T3D-TD appeared to induce RIG-I-dependent ISG expression, whereas the rapidly replicating T3D-PL appeared to induce RIG-I-independent genes. The role of any host gene on enhancing or inhibiting T3D reovirus replication was not examined in detail; thus, a large data set is presented but the impact or inhibitory mechanisms were not further explored.

The rationale at the beginning of this study seems to be to examine the reason for faster replication kinetics of T3D-PL, and to tie that to its oncolytic properties, but that idea never comes to fruition. In fact, previous studies already show that the replication kinetics are cell-independent. Therefore, it is unclear if any of the cell signaling responses described in this manuscript directly influence the T3D viral life cycle or its oncolytic properties.

Part II – Major Issues: Key Experiments Required for Acceptance</br>Please use this section to detail the key new experiments or modifications of existing experiments that should be <u>absolutely</u> required to validate study conclusions.

Generally, there should be no more than 3 such required experiments or major modifications for a "Major Revision" recommendation. If more than 3 experiments are necessary to validate the study conclusions, then you are encouraged to recommend "Reject".

Reviewer #1:

1. Although the authors showed some data in other mouse cell lines (B16-F10 and NIH3T3) in Fig 6, all the data presented is representative of a single mouse cell line, L929. The authors claimed that the study presented herein could be informative for the oncolytic reovirus community and the implementation of better oncolytic reoviruses for cancer treatment in a future. However, most of the data is generated in L929 cells, which are normal mouse fibroblasts, there is not data in human cancer cell lines. These are issues that would affect the translational impact of the findings, I do believe that lack of data related to this does limit somewhat the impact of the study"

Although L929 cells are typically referred to as immortalized normal fibroblasts, they were characterized as tumorigenic in mice [1-3], are highly permissive to reovirus, but importantly were previously used in our panel of human and mouse cancer cells lines and represented common phenotypic differences between T3D strains [4, 5]. Nevertheless, we completely agree that panels of cancer cells are required to understand the applicability of conclusions to diverse cancer cell backgrounds; and therefore we now attached our additional data showing reproducibility of key findings from our study in A549 cells (human lung carcinoma), B16-F10 (murine melanoma) and ID8 (murine ovarian cancer). Importanty, our previously demonstrated differences of T3D strain oncolysis in vivo used the B18-F10 melanoma model [4, 5], so the data we currently show in B16-F10 cells has extended relevance.

Lines 144-146 now describe L929 better: "Phenotypes were first characterized in tumorigenic L929 mouse cells (31-33), since our previous comparisons found that enhanced oncolytic properties of T3DPL in L929 cells was representative of a panel of human and mouse cancer cell system (19, 20). "

Lines 164-168 describe other cancer cell lines: "Increased phosphorylation of IRF3 by T3DTD relative to T3DPL was reproduced in A549 human lung carcinoma cells (Figure S1A-C). Increased induction of IFN/ISGs by T3DTD was further confirmed in ID8 murine ovarian cancer cells, and in the B16-F10 murine melanoma system where T3DPL was previously characterized to exert more oncolytic activity in vivo relative to T3DTD (Figure S1D, E)."

Lines 410-413 also describe other cells that reproduce the key conclusions: "Most surprising about the findings, is that RIG-I/IFN-dependent versus independent signalling were inversely activated by T3D laboratory strains, which held true in five cell lines evaluated: NIH/3T3 (Figure 7C, n=2), L929 (Figure 7D, n=3), MEF (Figure 5A versus S1F, n=2), B16-F10 (Figure S1D , n=2) and ID8 (Figure S1E, n=1). The data indicates that reovirus strains can differentially active both IFN/RIG-I-dependent and independent genes, and that minor virus genomic diversity has potential to induce distinct host gene and cytokine profiles.

2. Fig 1. The figure panels are not properly indicated in the text: Line 154 (should say Fig 1D), line 158 (should say Fig 1E) and line 159 correspond to Fig 1F.

Thank you, Corrected

3. Fig 1 legend (line 1090). Was the western blot performed with a μ2 or a μ1 antibody? There is a discrepancy between text and figure.

Thank you, Corrected

4. Fig 2B-C. Are there other factors in the genome of T3DPL important for the observed phenotype. How do the double and triple-reassortants perform in these qPCR assays?

My sincere apologies if I am misunderstanding this question. If the reviewer is asking how mutations in S4, M1 and L3 affect reovirus, then we describe in the manuscript what we currently know from our previous studies on reovirus replication kinetics [4, 5], which suggest that the M1-encoded u2 protein enhances transcription rates of the virus (even in a test-tube assay), L3-encoded l1 protein increases the size of virus factories. In the current manuscript, we find that the S4 polymorphism affects the induction of RIG-I/IFN-independent signalling. It seems that each of these affects, incrementally affects the kinetics of virus replication, and can be additive.

5. Fig 2D and line 197. It is hard to conclude that the mono-ressortant strains have an intermediate phenotype (especially if the plaque assay the dilution factor used was 1:10). The variation observed between the ressortants and the parental T3DTD could be just dues to titer error. Quantification of viral genomes by qPCR could be more informative. Also, stats and error bars should be indicates.

The differences in titers is most easy to appreciate at sub-saturation, so at 12hpi, and so the current figure 2D shows the range most easily. The intermediate replication phenotype of the mono-reassortants was strongly established in our previous studies [4, 5] , so we focused on getting multiple independent experiments for the IFN/ISG production, and also doing new experiments such as the CHX and GuCl ones to directly implicate virus replication kinetics in IFN induction levels.

6. Fig 2 legend. P values are not indicated.

Thank you, we have now described the one-way ANOVA with Dunnett's multiple comparisons test, * $p \leq 0.05$, *** $p \leq 0.001$, **** $p \leq 0.0001$, ns > 0.05

7. How does T3DPL "performs" in cells that have been pre-treated with type I interferons?. In vivo, for example in a tumor, it is expected that the main source of interferons indeed are not the infected cells but the immune cells that react to the infection.

We addressed this question with a new experiment. We tested the effects of adding recombinant IFN to the media, both pre- and post- infection. This new experiment suggests that IFN only minimally exerts inhibitory effects on virus replication when added after virus entry and uncoating is complete, but is strongly inhibitory in advance.

Lines 340-352 now describe: "As a third complementary strategy to assess the impact of IFNs on the first round of T3D reovirus replication, we treated L929 cells with IFNβ at various timepoints pre- and post- reovirus infection, and monitored cytokine expression (Figure S1A), virus titers (Figure S1B), or virus protein expression (Figure S1C) at 18hpi. We first validated that IFNβ treatment for 5hrs, in absence of virus, causes potent activation of prototypic ISGs (Cxcl10, Rsad2, Mx1), but not non-IFN-induced cytokines Ifnb1 itself and Cxcl1 (Figure S2A). In congruence with the paracrine effects of IFNβ on suppressing reovirus cell-cell spread in Figure 5, when cells were pre-treated with IFNβ for 5hrs prior to reovirus exposure (-5), there was strong inhibition of burst titers (Figure S1B) and protein synthesis (Figure S1C). To mimic potential autocrine effects of IFNβ on already-infected cells, recombinant IFNβ was added 3 or 5 hours post reovirus exposure. Effects of IFNβ on viral protein and titer accumulation was clearly diminished over time of infection, with minimal (not significant) effects by 3-5 hours post-infection for both T3DPL and T3DTD. Therefore, addition of IFNβ at timepoints following reovirus entry and uncoating had nominal impact on virus replication."

8. Please clarify the point made in lines 335-337. I thought that T3DPL was a better oncolytic, but herein you imply that T3DTD is better because induces interferon which can lead to tumor clearance?

We agree the this is an interesting question, and points to the value of our findings. We describe in Lines 477-495 that T3D strains clearly induce distinct cytokines, and have distinct oncolysis in vivo, but that one cannot directly relate a given cytokine and imply it affects oncolysis without further analysis. We suggest that in future, perhaps even a mixture of T3D strains, to induce a mixture of cytokines, might be ultimately beneficial. Specifically, we state "Reovirus-mediated oncolysis is a combination of both direct virus-induced cytolysis (49-53) and anti-tumor immune stimulation (54-59). Given that cytokines, chemokines, and innate signalling molecules strongly influence anti-tumor immunity (60-64), we predict that they will impact anti-tumor immunity during reovirus oncolysis and contribute to the immunotherapeutic value of reovirus. Our data provokes a close investigation into the roles of RIG-I/IFN-dependent versus independent cytokines on reovirus-strain-specific anti-tumor activity. Based on the 5 cytokines we analyzed, it is impossible to predict the impact of the distinct cytokine profile induced by each strain. As an example, CXCL10 is proposed to have both pro- and anti-proliferative action on Breast cancer depending on the receptors it engages (65). Most importantly, many more IFN-dependent and -independent cytokines are likely upregulated differentially by reovirus strains that were not evaluated in this study. A systematic approach will be necessary to determine precisely the benefits or costs of using reovirus strains that induce RIG-I/IFN-dependent versus independent cytokines. Should both groups of cytokines prove important during oncolytic virus treatment, then combination therapy with both T3DPL and T3DTD reovirus strains (or a hybrid variant that induces both types of signalling) warrants consideration. One must also consider that such cytokines would contribute to heightened clearance of the virus, and therefore to achieve a balance between strong virus replication and strong anti-tumor activity, immunologically distinct oncolytic viruses may need to be sequentially used. Overall, our findings suggest that apart from adding genes to oncolytic viruses to stimulate immunity, small genomic differences among virus strains could impact the immunotherapeutic potential of an oncolytic virus."

9. Fig 5. The data shown in panel C seems in disagreement with the data shown in panel H. MEF-DKO show lower T3DPL S4 levels compared to WT MEFs, but in panel H, RIG-I KD cells show higher T3DPL S4 levels compare to control.

The differences pointed out by the reviewer are minimal; specifically, the T3D-PL S4 mRNA difference between WT MEF and DKO MEF was ~1.3 whereas the difference between shRIG-I and shSCR was ~1.2. We therefore do not consider this a disagreement, but rather a suggestion that the difference is unlikely to be significant.

10. In the experiment where co-infections between T3DPL and T3DTD viruses were performed, how we can ensure that indeed "co-infections" happened and that the signalling events triggered independently by these two viruses indeed happen in "parallel'. I think the robustness of the conclusions and model proposed could strongly benefit by performing single cell sequencing.

Single cell sequencing is beyond the scope of this manuscript, and would not be necessary to draw the various conclusions. However, undoubtedly, a very cool technique we are excited to apply to future research.

11. Why T3DPL does not activate IRF phosphorylation and IFNs?

The data actually shows (and we describe) that T3D-PL does actually activate IRF-3 phosphorylation and expression of IFN and ISGs, but to a significantly lower extent compared to T3D-TD (Figures 1D, 1F, 1G, 2B, 2C, 3C, 3D, 4A, 4D, 5A, 5F, 6C, 6F.) But if the reviewer is curious why the difference, the data in the manuscript supports that that slow replication of T3D-TD leads to higher detection of the virus. We discuss a possible model, where if a virus doesn't establish infection fact enough, it doesn't "hide" viral PAMPs in dsRNA sequestering de-novo-synthesized sigma 3, or in factories, and easily detected. Since incoming reovirus particles initially establish distinct replication centers (factories), even during co-infections, a slow-replicating virus may reveal PAMPs and stimulate IFN production. Hopefully this re-cap of our manuscript helps address this question.

12. Indicate in all figure legends number of biological replicates and error bars and statistics have to be included when appropriate.

Thank you, Corrected

------REVIEWER 2-------

Reviewer #2: One overarching concern not addressed in the methods section, or in the experiments themselves, is the variability of different preparations of virus stocks. There is precedent for considerable variability in the immunoreactivity of different reovirus stock preparations, particularly with regard to variations in the particle:PFU ratio. The authors tangentially mention the effects of defective virions in activating innate immune signaling in both the introduction and discussion, but do not examine these concerns themselves. If, for example, the T3D-TD preparations have higher levels of defective particles that could deliver PAMPs to PRRs, that could account for greater innate immune signaling despite overall lower levels of viral RNA synthesis in the infected cell. A robust examination of the particle:PFU ratio of the stocks used would allay these concerns; similarly, if the experiments were repeated across multiple viral preparations, this would strongly suggest that it is a phenotype related to the cell biology of the infection, rather than the result of the initial inoculum. Evidence from Stuart et al also suggests that differential delivery of genomic dsRNA (possibly via differences in capsid stability during uncoating) underpins differences in IRF-3 activation, which is not addressed by the authors.

We thank the reviewer for this suggestion. The particle:pfu ratios for T3DPL and T3DTD parental viruses and respective S4, M1 and L3 gene reassortant viruses were calculated and have been included as a part of Figure 2A. Accompanying methods for determining particle:pfu ratios have been included in the materials/methods section. Importantly, we have elaborated to explain that pfu:particle ratios have minimal impact on IFN induction by reassortant viruses with respect to the parental viruses. Specifically, Lines 188-195 state: “To explore the possibility that differential levels of defective virions in the virus preparations could impact IFN induction, we compared specific infectivity of the viruses. The ratio of total particles versus infectious particles was determined using spectrophotometry at OD260 and standard plaque assay, respectively (Figure 2A, values below plaque photographs). There was no clear relationship between the specific infectivity and IFN induction (Figure 2A versus 2B); therefore, increased IFN induction could not be easily explained by an increased proportion of defective or non-productive virions. “

A second critical question is the location of virus factories in co-infected cells; the authors postulate a model whereby each entering core sets up its own virus factory, thereby keeping the different strains “segregated” in the cytoplasm, which facilitates i) the rapid replication of T3D-PL in its factories and ii) the detection of T3D-TD PAMPs in its factories. However, this model would tend to preclude the possibility of the production of reassortant viruses. While directly testing this (perhaps using FISH probes specific to distinct viral polymorphisms?) might be outside the scope of the current manuscript, directly demonstrating that the two virus strains elicit distinct factory compartments would certainly increase the overall significance of the work.

The reviewer is asking a very fun question, that we considered thoroughly – if viruses establish distinct factories (which others have shown for incoming reovirus cores [6, 7]), then how is there reassortment? The virions that enter a cell can establish separate replication centers around them, but over time, everything moves towards the perinuclear region where all viruses have opportunity to mix. We are now proposing a new idea based on our data, that during the initial “starting” replication centers, the incoming virions can exhibit distinct effects on the same cell depending on the genetics of the incoming virus. So, in a co-infection, one strain of reovirus can induce RIG/IFN signaling while the other does not. Yet eventually, both strains will mix and can re-assort; but the mixing is beside the point of RIG-I/IFN induction and beyond the scope of this manuscript.
We now address this in the discussion, specifically lines 535-541 describe “. It should be noted however, that virus factories/inclusion bodies eventually converge into a large perinuclear body; since we and others can easily generate reassortants during co-infection, the overall model needs to consider that incoming strains, although exerting distinct effects on IFN induction early, eventually mix and exchange segments.”

The idea of FISH (and other approaches) to further understand virus factory dynamics – is well within our curiosities and if the reviewer also works on similar questions, please collaborate with us! As the reviewer points out, it truly would be its own manuscript.

A final major suggestion would be to differentiate between plus- and minus-strand viral RNA in measuring levels of RNA synthesis in the experiments; the authors use randomers to facilitate cDNA synthesis, which presumably would equivalently amplify both viral mRNA as well as negative-sense genomic RNA. Using a reovirus specific primer (either forward or reverse to amplify negative-sense or positive-sense RNA respectively) would allow for the more fine-grained analysis of whether the differences between strains in RNA production is solely in producing mRNAs vs. producing genomic mRNA during the production of viral progeny particles.

We have previously used positive- versus negative- sense RNA primers for cDNA synthesis, when comparing reovirus kinetics,

but they always give the same trend. If the reviewer is not in the reovirus field, then I will try to explain why: The virus comes in with dsRNA, and synthesizes positive-sense. But positive sense very quickly serves as genome for re-encapsidation and creation of new “transcriptionally active cores” that generate negative sense within them, and then pump more positive sense into the cytoplasm. The process must be exceptionally logarithmic, because positive and negative senses amplify at similar trend to each other. For example, a slow-replicating T3D will have slower kinetics of both positive and negative, but the positive and negative will follow the same kinetics. The only time we see a difference between positive and negative, is when we artificially prevent negative sense production, for example by adding CHX or GuCl.

------REVIEWER 3-------

Reviewer #3:
1a. When evaluating IFN or ISGs by RT-qPCR, it is most common to set levels relative to the mock sample. By doing so, it is easier for the reader to evaluate and understand if levels have increased during viral infection. In this manuscript, the authors have chosen to set all samples relative to T3D-TD infection at MOI 1 – please provide an explanation for doing so. 1b. In setting T3D-TD at MOI 1 as the reference, it becomes difficult for the reader to determine how much higher levels of IFN or ISGs were in comparison to mock. In this situation, the authors must be exceedingly careful as to the conclusions being drawn. For instance, it is reasonable to state that in T3D-TD infected cells, the levels of Ifnb1 at MOI 9 are about 7-fold higher than at MOI 1, but from the data presented it cannot be determined if there is a measurable reduction in Ifnb1 levels comparing T3D-TD at MOI 1 to T3D-PL at MOI 1 (Fig. 1F). The authors then conclude that T3D-TD induced higher expression of ISGs Mx1 and Rsad2 relative to T3D-PL (line 160), yet Fig. 1G appears to show a less than 2-fold change in Rsad2 and Mx1 levels. The suggestion that T3D-TD triggered greater IFN-response signaling pathways (lines 161-162) does not appear to be supported by the data presented in Fig. 1G.

Thank you for the suggestion. Wherever possible, we converted data for mock to be 1. In few cases, however, when mock Cts are too high or unachievable, due to such small mRNA levels, we were forced to have other comparatives but always indicated our strategy in the figure legend. This should address the reviewers consideration of the data, for example figure 1E-G, where differences are significant at matched MOI between T3D strains.

2a. Although there is a complex attempt to show that IFN did not affect the first round of T3D-TD replication, the most direct experiment would use IFN treatment (pre-infection and/or post-infection) to show that IFN does not impact the initial round of T3D-TD replication. 2b. The authors suggest that the slower rate of T3D-TD replication causes greater IFN and ISG production. Therefore, there is an examination of mRNA levels of numerous ISGs, but there is no direct demonstration that any of them impact T3D-TD titers or spread. Which of the examined ISGs are relevant to the reovirus system? Do any of them inhibit T3D-TD or T3D-PL spread from cell to cell?

Additional experiments were completed and added to the manuscript to address this important point:

1. We now provide data showing that both viral mRNA levels (previously included), and now virus burst size (new data), are not increased in DKO cells, supporting further that IFN does not impinge on the first round of replication.

We now describe in lines 299-319: “A direct and physiologically relevant approach to implicate a pathway in virus replication is to remove the pathway and determine if virus replication is changed. Accordingly, we made use of double knock-out (DKO) mouse embryo fibroblasts (MEFs) lacking both RIG-I and MDA5 (41, 43). DKO cells were confirmed to show minimal induction of IFNs (Ifnα4) and IFN-induced gene Rsad2 following 12 hours of infection by either T3DPL or T3DTD (Figure 5A). We reasoned that if IFN signalling can impact the first round of virus infection, then the DKO cells should demonstrate increased infection by reovirus at an intermediate timepoint of 12-18hpi where cell-cell spread is minimal. Wild-type (WT) and DKO MEFs were exposed to equivalent infectious dose of T3DPL or T3DTD. The percent of cells productively expressing viral antigens was measured by flow cytometric analysis (Figure 5B) or visually monitored by fluorescence microscopy (Figure 5D, left). S4 reovirus mRNA production was quantified at 12hpi by RT-qPCR (Figure 5C). Virus input versus burst size were assessed by standard plaque titration (Figure 5D). By all three parameters of reovirus replication, when focusing on timepoints prior to cell-cell spread (≤18hpi), T3DPL outperformed T3DTD in the first round of infection as was anticipated. Most critically, knock-out of IFN production had minimal effects on either T3D strain during the initial round of replication. Note that IFN production did, however, exert the expected paracrine inhibitory effects on dissemination and subsequent rounds of reovirus replication, evident by increased numbers of infected cells (Figure 5B, 24hpi), titers (Figure 5D, 48hpi), and even plaque size (Figure 5E) in DKO relative to WT cells at time points ≥24hpi . The data was suggesting that T3DTD, via slower replication kinetics, causes greater IFN signalling; rather than that greater IFN signalling causes slower T3DTD replication kinetics. Moreover, while differences in IFN signalling are not responsible for delayed replication kinetics of T3DTD, they are likely to favor enhanced cell-cell spread for T3DPL over multiple rounds of infection.”

2. As suggested by the reviewer, we tested the effects of adding recombinant IFN to the media, both pre- and post- infection. This new experiment suggests that IFN only minimally exerts inhibitory effects on virus replication when added after virus entry and uncoating is complete.

Lines 340-352 now describe: “As a third complementary strategy to assess the impact of IFNs on the first round of T3D reovirus replication, we treated L929 cells with IFNβ at various timepoints pre- and post- reovirus infection, and monitored cytokine expression (Figure S1A), virus titers (Figure S1B), or virus protein expression (Figure S1C) at 18hpi. We first validated that IFNβ treatment for 5hrs, in absence of virus, causes potent activation of prototypic ISGs (Cxcl10, Rsad2, Mx1), but not non-IFN-induced cytokines Ifnb1 itself and Cxcl1 (Figure S2A). In congruence with the paracrine effects of IFNβ on suppressing reovirus cell-cell spread in Figure 5, when cells were pre-treated with IFNβ for 5hrs prior to reovirus exposure (-5), there was strong inhibition of burst titers (Figure S1B) and protein synthesis (Figure S1C). To mimic potential autocrine effects of IFNβ on already-infected cells, recombinant IFNβ was added 3 or 5 hours post reovirus exposure. Effects of IFNβ on viral protein and titer accumulation was clearly diminished over time of infection, with minimal (not significant) effects by 3-5 hours post-infection for both T3DPL and T3DTD. Therefore, addition of IFNβ at timepoints following reovirus entry and uncoating had nominal impact on virus replication.”

3. Several figures are lacking statistical analysis, which will be important to supporting some of the claims in the manuscript. Figs. 1E, F, G absolutely require statistical analysis. Fig. 2D is lacking error bars and statistical analysis. Fig. 5 and Fig. 7 are also lacking statistical analysis.

As correctly suggested by the reviewer, all figure legends now include details on the experimental replicates and specific statistical analysis. All key findings contain 3 or more independent experimental replicates with appropriate statistical analysis. Supportive findings using the shRNA knockdown model system were analyzed from 2 independent experimental replicates; statistics was only completed when at least 3 independent experiments were applied.

4. The use of the word “replication” in this manuscript is rather vague. When discussing reovirus infections, typically replication refers to the specific step of dsRNA synthesis. However, in this manuscript replication appears to refer to the entire life cycle from entry to nascent virion production. It would offer some clarity for more specific terminology to be used.

I appreciate the insight into other virologist perspectives. We have ensured that we say “viral replication” instead of “genome replication”, where the latter refers to genome copy. We are using “virus replication” or “virus replication cycle” to refer to the whole cycle, similar to how it is commonly used (for example, https://www.immunology.org/public-information/bitesized-immunology/pathogens-and-disease/virus-replication , https://en.wikipedia.org/wiki/Viral_replication, https://www.ncbi.nlm.nih.gov/pmc/articles/PMC7173495/,)

Part III – Minor Issues: Editorial and Data Presentation Modifications</br>Please use this section for editorial suggestions as well as relatively minor modifications of existing data that would enhance clarity.

Reviewer #1: Minor comments

Line 64- did the authors meant “it is not surprising”?
Context reads correctly as is.

Line 139- impact word used twice
Corrected: Given the possible impact of IFN production

Line 205- should say “production”
Corrected: The inverse relationship between IFN production

Line 240-typo (phosphor-IRF3)
Corrected: The higher phosphorylated-IRF3 levels

Line 331- the authors meant: “T3D PL (not T3DTD) inherently replicates better than T3DTD?
Corrected: In other words, T3DPL inherently replicates better than T3DTD

Line 383- Typo “Were strongly..”
Corrected: Surprisingly, Cxcl1, Csf2, Cxcl2, and Fas mRNAs were strongly induced

Reviewer #2:
1) Line 76 should read “burst size” not “bust size”
Thank you! Corrected: leading to higher burst size

2) Line 87 should read “distinct” not “district”
Corrected: interferon responses to distinct reovirus serotypes

3) Line 158-160: References to specific figure 1 panels in the text do not match with the specific panels in the figure
Corrected

4) Line 326: Do the authors mean that "IFN signaling does NOT account…"?
Corrected: that IFN signalling does not account

Reviewer #3:
1. There is strong support for replication of data from previous studies, but Fig. 1C and 2A appear to be replicated data previously published by the Shmulevitz lab. In a data-dense paper such as this one, it is okay to just refer to the previous studies to reduce the amount of data presented. Additionally, drawings representing well-known signaling pathways are not necessary to include.
Corrected to only include new and relevant that we felt the reader would need to see to draw conclusions, without forcing them to other manuscripts which is unfair. Importantly, all data in this manuscript is newly generated and was not previously used.

2. Please note in the Materials and Methods if no template and no reverse transcriptase controls were included in every RT-qPCR setup.
Yes, all RT-qPCR experiments included both no template and no reverse transcriptase controls. Materials and methods have been corrected accordingly.
Corrected: All RT-qPCR reaction plates included negative controls; no template and no reverse transcription controls.

3. There are several instances of references to figures in the text that do not match: for instances lines 158-159 refer to Fig. 1D, but the data is found in Fig. 1F; another instance in line 160 refers to Fig. 1E but the data is found in Fig. 1G. Please carefully make sure all figures are correctly referenced in the text.
Corrected.

4. Line 331 reads, "…T3D-TD inherently replicates better than T3D-TD…" – please correct.

Corrected.

References cited in response

1. Rodriguez, T., Rengifo, E., Gavilondo, J., Tormo, B. & Fernandez, A. Morphologic and cytochemical study of L929 cell variants with different metastasizing ability in C3HA/Hab mice. *Neoplasma* **31**, 271-279 (1984).
2. Gavilondo, J. *et al.* Neoplastic progression evidenced in the L929 cell system. II. In vitro growth properties and biochemical characteristics of cell variants with different malignant behavior. *Neoplasma* **29**, 281-293 (1982).
3. Gavilondo, J., Fernandez, A., Castillo, R. & Lage, A. Neoplastic progression evidenced in the L929 cell system. I. Selection of tumorigenic and metastasizing cell variants. *Neoplasma* **29**, 269-279 (1982).
4. Mohamed, A. *et al.* Single amino acid differences between closely related reovirus T3D lab strains alter oncolytic potency in vitro and in vivo. *J Virol* (2019).
5. Mohamed, A., Smiley, J.R. & Shmulevitz, M. Polymorphisms in the most oncolytic reovirus strain confer enhanced cell attachment, transcription and single-step replication kinetics. *J Virol* (2019).
6. Broering, T.J. *et al.* Reovirus nonstructural protein mu NS recruits viral core surface proteins and entering core particles to factory-like inclusions. *J Virol* **78**, 1882-1892 (2004).
7. Broering, T.J., McCutcheon, A.M., Centonze, V.E. & Nibert, M.L. Reovirus nonstructural protein muNS binds to core particles but does not inhibit their transcription and capping activities. *J Virol* **74**, 5516-5524 (2000).
